# Assessment of Patient Safety Culture in a Pediatric Department

**DOI:** 10.7759/cureus.14646

**Published:** 2021-04-23

**Authors:** Mohammed Alsabri, Fiorella B Castillo, Salome Wiredu, Ahmad Soliman, Tracy Dowlat, Viswanathan Kusum, Fernanda E Kupferman

**Affiliations:** 1 Pediatrics, Brookdale University Hospital Medical Center, Brooklyn, USA; 2 Pediatrics, Brookdale University Hospital Medical Center, brooklyn, USA; 3 Clinical Risk Management and Corporate Compliance, Brookdale University Hospital Medical Center, Brooklyn, USA; 4 Pediatrics, Brookdale University Hospital Medical Center, Brooklyn , USA

**Keywords:** pediatrics, patient safety culture, usa

## Abstract

Background

An assessment of the prevalent culture needs to be the first step when building patient safety programs in healthcare organizations to achieve high-quality health care.

Objective

To conduct a baseline assessment of patient safety culture, to provide insight into the factors that contribute to patient safety, and to use the information to make improvements.

Methods

The Hospital Survey on Patient Safety Culture (PSC) questionnaire was conducted from October through December 2020 at the Brookdale Hospital Medical Center (BHMC) Pediatric departments (Pediatric Inpatient Unit, Neonatal Intensive Care Unit [NICU], Pediatric Intensive Care Unit [PICU], and Pediatric Emergency Department) and four community-based ambulatory pediatric practices (Brookdale Family Care Centers [BFCC]). The percentages of positive responses on the 12 patient-safety dimensions and the summation of PSC and two outcomes (overall patient safety grade and adverse events reported in the past year) were assessed. Factors associated with PSC aggregate score were analyzed.

Results

From the 385 emails that were sent, 136 surveys were considered for analysis. This gives us a response rate of 35.3%. Most of the participants were nurses (58%) with direct contact with patients (94.2%). Most respondents did not report any events (60.7%), whereas 30.3% reported 1-2 events in the past year. The patient safety composites with the highest positive scores were teamwork within units (78%), supervisor/manager expectations and actions promoting patient safety (71.2%), and organizational learning--continuous improvement (66.8%). The composites with the lowest scores were non-punitive response to error (35.9%) and staffing (38%).

Conclusions

All of our composite measures, with the exception of teamwork within units, appear to be low, which means that all the other composite measures require interventions for improvement of overall safety culture. In order for healthcare leaders and policymakers to establish a culture of safety and improvement, they must create a climate of open communication, continuous learning, and eliminate the fear of blame and punitive feedback.

## Introduction

Patient safety is defined by the Institute of Medicine (IOM) as ‘the freedom from accidental injury due to medical care or medical errors’ [1]. The issue has received significant attention following the release of the renowned report from the IOM, ‘To Err is Human: Building a Safer Health System’ [1]. The main message in the report was that preventing death and injury from medical errors requires dramatic and system-wide changes [2].

Patient safety and Patient Safety Culture (PSC) are becoming areas of increasing interest in healthcare. Safety culture has been defined as the product of individual and group values, attitudes, perceptions, competencies, and patterns of behavior that determine the commitment to, and the style and proficiency of an organization's health and safety management [3]. Developing a positive patient safety culture is a crucial element in the improvement of patient safety in a healthcare organization [4- 5]. Achieving a culture of patient safety requires an understanding of the values, beliefs, and norms about what is important in an organization, and what attitudes and behaviors related to patient safety are supported, rewarded, and expected [6].

The assessment of the prevalent culture is a first step that should precede designing patient safety programs in hospitals [7]. Employees with positive safety culture are more likely to engage in safety-related behaviors when compared to those with perceived negative safety culture [8]. The Agency for Healthcare Research and Quality (AHRQ) developed an extremely useful tool to assess healthcare organization culture regarding patient safety known as the Hospital Survey on Patient Safety Culture (HSOPSC) [7]. This tool has been widely used in different healthcare settings across many countries [9-11].

Some departments such as the Pediatric Intensive Care Unit (PICU), Neonatal Intensive Care Unit (NICU), and Emergency Department (ED) are considered high-risk environments for safety incidents [12]. Reasons include high patient volume, patient acuity, and complexity, a work environment characterized by time constraints, multiple interruptions, and disturbed sleep cycles for health care workers, as well as factors such as high-risk diagnostic and therapeutic interventions and variable levels of physician training [13-14].

It is globally understood that high-quality and safe patient care can only be provided if doctors are well prepared for this task through residency and teamwork training [14-15]. Without a doubt, the results will provide evidence to help relevant decision-makers and stakeholders within the healthcare system to build up effective strategies that may help improve the quality of care and ensure patient safety [16-17]. With the collective goal of improving the safety of care provided to patients, quality improvement initiatives that elucidate factors to improve safety culture and support a positive safety culture must be considered [18-19]. These efforts require concerted work and collaboration from hospital leaders and front-line staff to prospectively address any care limitations through systems-based solutions [18-19-20].

To date, there is no study understanding the safety culture and contributing factors at the pediatrics department of the Brookdale Hospital Medical Center (BHMC). Brookdale Hospital Medical Center is a community hospital that serves Central and Eastern Brooklyn, New York City -- an area with high rates of poverty, crime, and substance use. The majority of residents are Black/African American (approximately 71%) and 37% of residents are foreign-born. Brookdale serves a patient population in East Brooklyn that is predominantly low-income, with at least 50% receiving government income support (Temporary Assistance for Needy Families, Supplemental Security Income, and Medicaid). Most patients come from neighborhoods with a large concentration of poverty (average median household income of $32,000). Approximately 32% of residents live below the federal poverty level, compared to 22% for the borough of Brooklyn, and 19% for NYC overall. Patients are mainly insured through Medicaid and depend disproportionately on local safety net providers. Almost all the neighborhoods that Brookdale serves have the Health & Human Services (HHS) designations of Health Professional Shortage Area and/or Medically Underserved Area. Some of the factors that result in poor health outcomes for the population include high disease burden, lack of access to care, shortage of primary care doctors, linguistic and cultural isolation, and low health literacy. Using another measure of economic stress, the rent burden, and housing insecurity are high.

Brookdale Hospital and Medical Center is one of Brooklyn's largest nonprofit community teaching hospital with 530 beds serving the communities of Brownsville and East New York in Brooklyn. The department of pediatrics has an inpatient unit, pediatric emergency department, pediatric and neonatal intensive care units, and five ambulatory care centers in addition to a robust residency program. Therefore, this study aimed to conduct a baseline assessment of patient safety culture, to provide insight into the factors that contribute to patient safety, and to use the information to make improvements. 

## Materials and methods

Study design and setting

This is a cross-sectional study using the HSOPSC [6-21-22] conducted at the BHMC pediatric departments (Pediatric Inpatient Unit, NICU, PICU, Pediatric ED) and four community-based ambulatory pediatric practices (Brookdale Family Care Centers [BFCC]). Data was collected from October through December 2020.

Participants

The study targeted all the clinical and non-clinical staff in the pediatrics department who have direct contact with patients (physicians, nurses, patient care technicians, respiratory therapists), and staff without direct contact with patients but whose work directly affects patient care (pharmacists and supervisors, clerks, social workers). The research team contacted department managers and other department leaders to support this project by providing staff names, emails and promoting the survey during staff meetings [7]. All targeted staff available throughout the study period and across all shifts were contacted for study participation; a second email and a third email were sent as a reminder within an interval of 2-3 weeks in between. We followed the AHRQ guidelines which proposed to examine each returned survey for possible evaluation before the survey responses are entered into the dataset. We excluded surveys that were completely blank or contained responses only for the background demographic questions or contain the same answer to all the questions in the survey. Since a few survey items are negatively worded, the same response to all items indicates the respondent probably did not pay careful attention and the responses were probably not valid.

Exclusion Criteria

1- Staff on administrative or extended sick leave.

2- Staff who has moved to another hospital area/unit.

3- Staff without a BHMC email account. 

Evaluation tool and data collection

The HSOPSC was used to assess the PSC within the pediatrics department. The HSOPSC is the survey instrument that was used to collect the data. The HSOPSC was developed for the AHRQ to obtain a better understanding of the PSC of an entire hospital or of specific departments. HSOPSC has been primarily used for intra- and inter-institutional comparisons [21-22]. The HSOPSC is based on a set of pilot studies carried out in 21 different hospitals across the US, involving 1,461 hospital staff. The HSOPSC consists of 42 items grouped in 12 composites [18] and includes both positively and negatively worded items; a five-point Likert scale is used to score each item. For each item, we calculated the mean score, standard deviation (SD), and percent positive scores; we reverse-coded negatively worded items (% of items receiving a score of 4 or 5 for positively worded items and a score of 1 or 2 for negatively worded items). Accordingly, areas of strength in PSC were defined as “those items that received positive answers from 75% of respondents,” whereas areas of potential for improvement were identified as “having potential for PSC improvement received negative answers from 50% or more of respondents.” To calculate the composite scores (CS), we summed the items within the composite scales and divided the sum by the number of items. Moreover, an aggregate score was computed by adding up all the CS and dividing by the number of items.

Additionally, we added two single-item outcome measures, the overall patient safety grade (rated as “excellent, very good, acceptable, poor, and failing”) and the number of adverse events reported last year (rated as “No events, 1-5 events, and > 5 events”). Cronbach’s α was used to calculate internal consistency. Based on previous studies; values ≥ 0.6 are considered to have good reliability.

Ethical considerations

The project was conducted in an ethical and confidential manner. The researchers obtained BHMC Institutional Review Board (IRB) approval prior to carrying out the survey. The questionnaires were conducted anonymously and collected exclusively for research purposes. A written consent form was included on HSOPSC letterhead and included information about study objectives provided to each participant. Participants were asked for their consent by asking them to answer the questions only if they agreed. Participation was voluntary with the right to withdraw at any time. No personally identifiable information (such as the name of the respondent, email address, or Kronos number) was collected through the survey or field observation. The electronic survey doesn’t have any identifiable information linking the electronic data. Any surveys that have inadvertently included names or other identifying information were immediately excluded.

Statistical analysis 

This study used Statistical Package for Social Sciences (SPSS) software, version 26 (IBM Corp, Armonk, NY) for statistical analyses. We calculated descriptive statistics for the demographic characteristics and the study variables as well as the percentage of positive responses for each composite. Negatively worded items in the HSOPSC were reverse coded. The differences between background characteristics and reported patient safety culture among the different staff groups were examined by χ2 tests and one-way ANOVAs. The relationship between the explanatory variables (seven background variables and 12 dimensions of patient safety culture) and the outcome variable (overall patient safety grade) was examined by bivariate and multivariate logistic regression. Patient Safety Grade was dichotomized into high (“excellent” and “very good”) and low (“failing” to “acceptable”) for this analysis. The level of statistical significance was set to < 0.05. The Hosmer-Lemeshow test (p>0.05) showed goodness-of-fit. Multicollinearity was checked by the variance inflation factor (VIF<3.357).

## Results

Respondents’ characteristics

The Cronbach’s α values for the 12 AHRQ composites ranged from 0.6 to 0.8 [6-23]. In this study, Cronbach's α values varied between 0.31 and 0.95 (Table 1). From the 385 emails that were sent, 136 surveys were considered for analysis. This gives us a response rate of 35.3%, which is adequate for this type of study and method of administration. Out of the 136 respondents that reported their position, 22.3% were nurses and licensed vocational nurses and licensed practical nurses (LVN/LPNs), 35.7% were physicians (attendings, residents) and physician assistant (PA)/nurse practitioner (NP), 3.2% were patient care technicians, 3.8% were unit clerks/secretaries, and the remainder were pharmacists, technicians, therapists, dieticians, and management (11.5%). From our respondents, 45% percent had professional experience of 11-15 years, and 28.33% had professional experience of 1-5 years. Most of the respondents had also worked for 11-15 years in the hospital (42.6%), and within their unit area for 1-5 years (44.6%). Thirty-nine percent worked 20 to 39 hours per week, 27.9% worked 40 to 59 hours per week, and 27.9% worked 60 to 79 hours per week. Moreover, about 94.2% of the respondents had direct interaction with patients. Most respondents did not report any events (60.7%), whereas 30.3% reported 1-2 events (Table2).

**Table 1:** Cronbach's alpha for the 12 HSOPSC composites HSOPC: Hospital Survey on Patient Safety Culture

Patient Safety Culture Composites	Cronbach's alpha
Teamwork Within Units	0.847
Supervisor/Manager Expectations & Actions Promoting Patient Safety	0.726
Org Learning--Continuous Improvement	0.796
Management Support for Patient Safety	0.888
Overall Perceptions of Patient Safety	0.742
Feedback & Communication About Error	0.799
Communication Openness	0.655
Frequency of Events Reported	0.956
Teamwork Across Units	0.807
Staffing	0.311
Handoffs & Transitions	0.902
Nonpunitive Response to Error	0.629

**Table 2:** Patient safety outcome variables by respondent characteristics *p ≤ 0.0001 **Other: pharmacist, dietician, respiratory therapist, physical, occupational, or speech therapist, technician (e.g., electrocardiogram, lab, radiology), administration/management PA: physician assistant; NP: nurse practitioner; RN: registered nurse; LVN: licensed vocational nurse; LPN:  licensed practical nurse

Respondent Characteristics		All Participants (N=122)		Attending/Physician/ Resident/PA or NP (n=56)		Patient Care Asst/Aide/Care Partner (n=5)		RN/LVN/LPN (n=35)		Unit Asst/Clerk/ Secretary (n=6)		Other** (n=18)		p	
		n	%	n	%	n	%	n	%	n	%	n	%		
Time Worked in the Hospital (Years) (n=122)															
	Less than 1 year	16	13.1%	12	21.4%	0	0.0%	4	11.4%	0	0.0%	0	0.0%	0.13	
	1 to 5 years	46	37.7%	24	42.9%	2	40.0%	10	28.6%	3	50.0%	7	38.9%		
	6 to 10 years	8	6.6%	1	1.8%	0	0.0%	2	5.7%	1	16.7%	3	16.7%		
	11 to 15 years	52	42.6%	19	33.9%	3	60.0%	19	54.3%	2	33.3%	8	44.4%		
Time Worked in Their Current Hospital Work Area/Unit (Years) (n=121)															
	Less than 1 year	17	14.0%	13	23.6%	0	0.0%	4	11.4%	0	0.0%	0	0.0%	0.33	
	1 to 5 years	54	44.6%	25	45.5%	3	60.0%	14	40.0%	3	50.0%	8	44.4%		
	6 to 10 years	8	6.6%	1	1.8%	1	20.0%	2	5.7%	1	16.7%	3	16.7%		
	11 to 15 years	42	34.7%	16	29.1%	1	20.0%	15	42.9%	2	33.3%	7	38.9%		
Time Worked in Their Current Specialty or Profession (Years) (n=120)															
	Less than 1 year	15	12.5%	10	18.2%	0	0.0%	4	11.8%	1	16.7%	0	0.0%	0.91	
	1 to 5 years	34	28.3%	16	29.1%	2	40.0%	7	20.6%	2	33.3%	7	38.9%		
	6 to 10 years	17	14.2%	7	12.7%	0	0.0%	5	14.7%	1	16.7%	3	16.7%		
	11 to 15 years	54	45.0%	22	40.0%	3	60.0%	18	52.9%	2	33.3%	8	44.4%		
Typical Hours Worked Per Week (Hours) (n=122)															
	Less than 20 hours per week	5	4.1%	5	8.9%	0	0.0%	0	0.0%	0	0.0%	0	0.0%	p≤0.0001*	
	20 to 39 hours per week	48	39.3%	10	17.9%	2	40.0%	22	62.9%	5	83.3%	9	50.0%		
	40 to 59 hours per week	34	27.9%	11	19.6%	3	60.0%	10	28.6%	1	16.7%	7	38.9%		
	60 to 79 hours per week	34	27.9%	29	51.8%	0	0.0%	3	8.6%	0	0.0%	2	11.1%		
	80 to 99 hours per week	1	0.8%	1	1.8%	0	0.0%	0	0.0%	0	0.0%	0	0.0%		
Interactions with patients (n=120)															
	Yes	113	94.2%	54	100.0%	5	100.0%	35	100.0%	5	83.3%	13	72.2%	p≤0.0001*	
	No	7	5.8%	0	0.0%	0	0.0%	0	0.0%	1	16.7%	5	27.8%		
Overall Patient Safety Grade															
	Mean (sd)	3.72	0.98	3.61	1.04	4.00	0.71	3.69	0.90	4.33	1.03	3.83	0.99	0.44	
Number of events reported (n=122)															
	No event reports	74	60.7%	34	61.8%	4	100.0%	15	45.5%	5	83.3%	12	66.7%	0.73	
	1 to 2 event reports	37	30.3%	15	27.3%	0	0.0%	13	39.4%	1	16.7%	6	33.3%		
	3 to 5 event reports	8	6.6%	4	7.3%	0	0.0%	4	12.1%	0	0.0%	0	0.0%		
	6 to 10 event reports	1	0.8%	0	0.0%	0	0.0%	1	3.0%	0	0.0%	0	0.0%		
	11 to 20 event reports	1	0.8%	1	1.8%	0	0.0%	0	0.0%	0	0.0%	0	0.0%		
	21 event reports or more	1	0.8%	1	1.8%	0	0.0%	0	0.0%	0	0.0%	0	0.0%		

Composites and area with potential for improvement 

**Figure 1:**
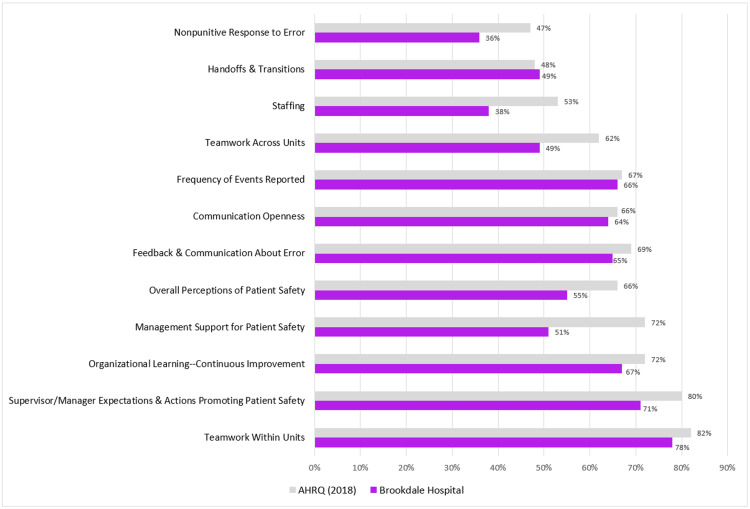
Comparison of the average positive response rate for each composite for Brookdale Hospital with AHRQ data AHRQ: Agency for Healthcare Research and Quality

A strength area (positive response rate >75%) was found in “teamwork within units” (78%). Scores below 50% were for “teamwork across units” (49%), “staffing” (38%), “handoffs and transitions” (49%), and “nonpunitive response to error” (36%) which need improvement. The average positive responses were generally lower than those for the AHRQ data [6-23], with the largest differences found in “management support for patient safety” (21% difference). “Handoffs and transitions” scored higher by 1% than the AHRQ data (Figure 1).” There were significant differences in the type of professionals across all the PSC composites (Table 3). The mean grade for overall patient safety across all participants was 3.7. While this rating did not significantly differ between staff positions, unit assistants, clerks and secretaries provided the highest overall patient safety grade (4.3). On the other hand, attendings, physicians, residents/PA, or NP gave the lowest grade (3.6) (Table 2). The bivariate analysis showed working in pediatrics for 1-10 years, reporting events, and the PSC composites of “Overall Perceptions of Patient Safety,” “Feedback & Communication About Error,” “Staffing,” and “Handoffs & Transitions” were significantly associated with overall patient safety grade (Table 4). Variables that were associated (p<0.20) with the target high overall patient safety grade in bivariable logistic regression models were considered as covariates for multivariable analyses. The findings for the multivariate analysis showed that having worked from 1-10 years in pediatrics (OR=10.8, p=0.03) and the patient safety composites of “Overall Perceptions of Patient Safety” (OR=1.0, p=0.001). "Feedback & Communication About Error” (OR=1.0, p=0.001), and “Handoffs & Transitions” (OR=1.0, p=0.014) were more likely and significantly associated with higher overall patient safety grade. However, having worked in pediatrics for >10 years, reporting events, and the PSC composite “Staffing” were not significantly associated with overall patient safety grade. Staff members who reported events were 62% less likely to provide a high patient safety grade, albeit this was not significant.

**Table 3:** Patient safety culture composites by type of staff *p<0.05 PA: physician assistant; NP: nurse practitioner; RN: registered nurse; LVN: licensed vocational nurse; LPN:  licensed practical nurse

Patient Safety Culture Composites		All Participants (N=120)		Attending/Physician/Resident/PA or NP (n=56)		Patient Care Asst/ Aide/Care Partner (n=5)		RN/LVN/LPN (n=35)		Unit Asst/Clerk/ Secretary (n=6)		Other (n=18)		p
		Mean	SD	Mean	SD	Mean	SD	Mean	SD	Mean	SD	Mean	SD	
1	Teamwork Within Units	78.3	5.9	75.4	4.4	90.0	20.0	80.4	8.5	70.0	6.7	83.3	7.9	0.002*
2	Supervisor/Manager Expectations & Actions Promoting Patient Safety	71.2	7.8	73.2	14.5	90.0	11.5	67.2	6.0	66.7	13.6	66.7	9.1	0.001*
3	Organizational Learning--Continuous Improvement	66.8	9.0	59.3	4.4	93.3	11.5	78.9	6.5	61.1	19.2	68.6	13.6	0.001*
4	Management Support for Patient Safety	51.6	8.1	51.2	10.7	86.7	23.1	51.0	6.1	55.6	9.6	40.7	8.5	0.002*
5	Overall Perceptions of Patient Safety	55.4	12.7	50.0	12.4	65.0	19.1	64.5	13.2	58.3	21.5	41.8	29.9	0.002*
6	Feedback & Communication About Error	64.9	9.6	61.6	4.9	80.0	20.0	60.0	15.9	61.1	9.6	73.4	13.4	0.001*
7	Communication Openness	64.3	11.0	50.9	5.5	100.0	0.0	84.2	3.2	83.3	0.0	59.5	11.2	0.001*
8	Frequency of Events Reported	65.6	1.7	61.9	9.8	70.0	17.3	72.4	11.6	55.5	25.5	63.0	17.8	0.001*
9	Teamwork Across Units	48.6	11.5	47.5	13.0	68.8	19.3	54.5	16.9	54.2	16.0	37.5	5.3	0.001*
10	Staffing	38.0	10.5	37.6	13.3	20.0	16.3	48.2	13.1	25.0	21.5	34.7	8.3	0.003*
11	Handoffs & Transitions	49.0	7.3	46.4	8.7	73.8	9.5	59.6	8.1	62.5	8.3	29.2	9.5	0.002*
12	Nonpunitive Response to Error	35.9	12.0	38.3	6.2	40.0	0.0	43.4	11.4	16.7	23.5	27.8	31.4	0.002*

**Table 4:** Bivariate and multivariate logistic regression models with overall patient safety as a response variable *p<0.05 OR: odds ratio; CI: confidence interval; PA: physician assistant; NP: nurse practitioner

	Bivariate Model				Multivariate Model			
	OR	95% CI		p	OR	95% CI		p
Time Worked in Their Current Specialty or Profession								
< 1 year (reference)								
1-10 years	20.90	1.40	311.71	0.03*	10.82	1.26	92.92	0.03*
>10 years	5.15	0.41	64.58	0.20*	5.93	0.82	42.91	0.08
Number of events reported: ≥1	0.25	0.04	1.41	0.12*	0.38	0.11	1.38	0.14
Staff Position: Attending/Physician/Resident/PA or NP	0.39	0.06	2.53	0.33				
Typical Hours Worked Per Week: >40 Hours	2.01	0.38	10.78	0.41				
Has Direct Patient Contact	6.35	0.08	492.45	0.41				
Patient Safety Culture Composites								
1. Teamwork Within Units	1.01	0.98	1.05	0.55				
2. Supervisor/Manager Expectations & Actions Promoting Patient Safety	1.01	0.98	1.04	0.69				
3. Organizational Learning--Continuous Improvement	1.01	0.98	1.04	0.47				
4. Management Support for Patient Safety	1.00	0.97	1.02	0.84				
5. Overall Perceptions of Patient Safety	1.03	1.00	1.06	0.04*	1.04	1.02	1.07	0.001*
6. Feedback & Communication About Error	1.03	1.00	1.06	0.06*	1.03	1.02	1.05	0.001*
7. Communication Openness	1.00	0.97	1.02	0.77				
8. Frequency of Events Reported	1.01	0.99	1.03	0.41				
9. Teamwork Across Units	1.00	0.96	1.03	0.80				
10. Staffing	0.97	0.93	1.00	0.05*	0.98	0.95	1.00	0.06
11. Handoffs & Transitions	1.03	1.00	1.06	0.05*	1.02	1.00	1.04	0.014*
12. Nonpunitive Response to Error	1.02	0.99	1.04	0.21				

## Discussion

This is the ﬁrst study tackling the issue of patient safety culture in the pediatric departments of the BHMC. With respect to the different composite measures, the response was poorest for the “non-punitive response to errors” with a positivity rate of response at just 36%. This is possibly attributable to the fact that studies have shown that punitive responses to errors are the main obstacle for disclosure of errors once they are identified [1-24]; a possible remedy to which is to establish a just culture [24] that recognizes errors as system failures rather than individual failures and encourages the staff to report events without fear of blame is essential for better error identification and continuous improvements. Patient safety improvements can only occur in learning organizations where preventive measures are taken after adverse events and near misses are identiﬁed, reported, and analyzed [25]. Therefore, under-reporting of events can hinder organizational improvement speciﬁcally regarding patient safety. Similarly, a study conducted by El-Jardali et al. revealed that a punitive response to an error is a major barrier for disclosure of errors upon their identiﬁcations [9]. The majority of respondents felt that their mistakes were held against them and later kept in their ﬁles. It is worthy to note that at our facility the mode or reporting of errors, incidents, and areas for improvement is via RL6 software (RL Solutions, Chicago, USA). This software seems to be somewhat underutilized at our facility, which could be due to the perceived punitive environment, however, further studies are needed in order to ascertain the exact reason(s).

Understaffing was the area receiving the penultimate amount of poor positivity scores, with scores much lower than the US data; the same was evident in a study by Hao et al. [26]. This is crucial as poor staffing/understaffing has been implicated in poor and adverse patient outcomes by a multitude of studies, so it is not surprising that hospitals have the consistent perception of poor staffing negatively affecting patient safety. It of course follows that with improved staffing, patient safety would congruently improve [24-26].

The areas with the highest positivity ratings are “teamwork within units” (78%) and “supervisor/manager expectations and actions promoting patient safety” (71%). This is consistent with the AHRQ’s (2018) trend. The high positivity rating on teamwork within units may speak to a culture of camaraderie and a sense of collective spirit within units as locally, within institutions, the relationship built within units may help foster teamwork. This was also appreciated in a PSC study of 44 NICUs by Profit et. al., which showed the highest scores reporting good teamwork within units followed by the overall perception of safety [27]. Ironically, however, teamwork across units has a positivity rating of 49%, which indicates that the intra-unit camaraderie does not transcend unit barriers or extrapolate to agency-wide teamwork. This may be an area that leadership may need to examine further in order to foster inter-unit teamwork and camaraderie.

“Supervisor/manager expectations and actions promoting patient safety” was also highly rated (78%), which possibly speaks to high input in terms of effort in leadership without payoff as the overall ratings in our agency are below the national standard. Some studies have shown that hospitals with fewer hierarchical and bureaucratic requirements have better overall scores [9,10,24]. This can be explained by the possible improved relations where there are less hierarchy and bureaucratic impediments.

The Cronbach’s α for our study components was indicative of high internal validity (0.6-1.0) for 11 composite measures. The only measure with a poor Cronbach’s α was “staffing” (0.3). Therefore, our study had good internal validity overall because according to the HSOPSC user’s guide, a Cronbach’s α of at least 0.6 delineates adequate internal validity. Referring to respondent characteristics, compared with the average composite scores only, respondent’s “typical hours worked per week” and “interactions with patients” were statistically significant (p≤0.0001). This is in keeping with the concept that the higher the amount of time spent and agency and highest interaction with a patient, the more adept a practitioner is to patient safety and the more credence their view of patient safety and PSC is [24,26,27]. 

Overall, our patient safety composite components have a lower positivity score compared to the AHRQ (2018) averages, and with the high internal validity, this is likely to be accurate which tells us that we ought to work to improve the components which are lagging, and institute policies which foster empowerment of staff to be active stakeholders in improving PSC. 

We recommend that future analysis ought to reexamine our agency in a future time period after the above recommendations are put in place in order to see the effectiveness (if any) of the applied interventions, a la Hao et al. did in Chinese hospitals which led to improved patient safety parameters and overall culture [26]. The conclusions of the aforementioned study were to recruit more employees, develop training programs for various positions, provide management support, and establish a just culture to promote a strong PSC in addition to regular assessment of safety culture [26]

Limitations

Our study limitations are mainly fourfold, consisting of an adequate yet low response rate, the effect of the coronavirus disease 2019 (COVID-19) pandemic, the slight over-representation of nursing and allied nursing staff in our study sample, and minor study technicalities/shortfalls. Our overall response rate was roughly 35.3% (n=136), which was significantly poorer than anticipated. The relatively small sample size could affect the generalizability of the results. Nevertheless, our results are similar to those of other comparable studies from other institutions, even if they were not conducted mainly in pediatrics departments. Another limitation is the impact of the COVID-19 pandemic because it effectively changed the working schedules and logistics of the working environment, which potentially affected our study, in terms of response rate and also possible added stressors in terms of patient safety given the fluid nature of changing directives and guidelines surrounding the pandemic, the added need of safety from personal protective equipment and isolations protocols, as well as patient care challenges in an uncertain environment. The results, therefore, should be interpreted cautiously, given the shadow of the COVID-19 crisis at the time of the study, which might have had an impact on the perceptions of the pediatric staff on PSC. Lastly, the distribution of a cross-sectional self-administered survey may also not have been the best methodology to assess PSC due to subjectivity.

## Conclusions

To conclude, all of our composite measures with the exception of teamwork within units was lower than 75% (acceptable standard set by AHRQ) positivity rating on the PSC questionnaire, which means that all the other composite measures require interventions for improvement of overall safety culture. The recommended interventions/changes based on our findings and that of other studies should be under the umbrella of decreasing perceptions of a punitive environment while fostering one which encourages reporting of errors and concerns surrounding patient safety as well as adequate staffing, thereby reducing a stressful, possibly error-prone working environment. It is vital for future reference that once the recommended interventions are implemented, this study is repeated in order to ensure that the implemented intervention have indeed been effective and further changes implemented if there are still shortcomings.
